# Examining the Effect of Ionizing Radiations in Ion-Exchange Membranes of Interest in Biomedical Applications

**DOI:** 10.3390/membranes13060592

**Published:** 2023-06-10

**Authors:** Íñigo Lara, Yago Freijanes, Sagrario Muñoz, Gema Ruiz, V. María Barragán

**Affiliations:** 1 Department of Structure of Matter, Thermal Physics and Electronics, Faculty of Physics, Complutense University of Madrid, 28040 Madrid, Spain; inigolar@ucm.es (Í.L.); smsm@ucm.es (S.M.); 2 Radiotherapy Service at the General University Hospital Gregorio Marañón, 28007 Madrid, Spaingruiz@salud.madrid.org (G.R.)

**Keywords:** ion-exchange membranes, reinforcement, ionizing radiation, swelling, kinetic, dimensional change

## Abstract

The possible effects of ionizing radiation on four commercial membranes, which are typically used as electrolytes in fuel cells supplying energy to a huge variety of medical implantable devices, were studied. These devices could obtain energy from the biological environment through a glucose fuel cell, which could be a good candidate to replace conventional batteries as a power source. In these applications, materials with high radiation stability for the fuel cell elements would be disabled. The polymeric membrane is one of the key elements in fuel cells. Membrane swelling properties are very important because they affect the fuel cell’s performance. For this reason, the swelling behaviors of various samples of each membrane irradiated with different doses were analyzed. Each sample was irradiated with a typical dose of a conventional radiotherapy treatment, and the regular conditions of the biological working environment were simulated. The target was to examine the possible effect of the received radiation on the membranes. The results show that the ionizing radiation influenced their swelling properties, as well as that dimensional changes were dependent on the existence of reinforcement, be it internal or external, in the membrane structure.

## 1. Introduction

Implantable medical devices in the body are one of the most commonly used therapies in various specialized fields to, for example, restore the heart rate, facilitate hearing, or relieve Parkinson’s symptoms. All implants have in common their small size, which avoids unwanted side effects, and their need for a continuous source of energy that allows their operation for a life time as long as possible. 

Most of these devices include very small batteries that can withstand various electromagnetic induction recharges. However, currently available bioelectronic implants consume too much power to be continuously operated on rechargeable batteries, and the end of these batteries’ lives always requires new surgery for users. The characteristics of our current society, with longevity and pathologies emerging at earlier ages, require a stable and autonomous source of energy, avoiding replacement-related surgical interventions and the risk that entails. This leads to the search for a battery that could replace conventional batteries and has the following main characteristics: economic value, rechargeability and biodegradability. The symbiosis of the search for biofuels, as an important part of the resources of our environment, and of circuits capable of generating electricity from biological fluids, directed research towards glucose fuel cells (GFCs) as an ideal solution to extract energy from the patient’s own biological environment without causing adverse physiological effects [1].

**Figure 1:**
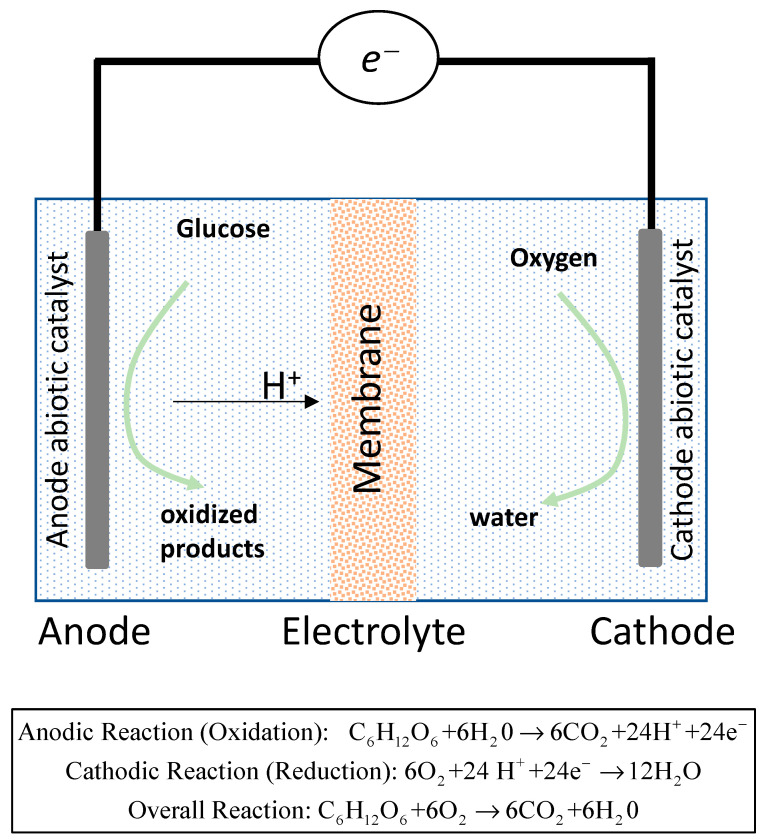
Schematic representation of an abiotic glucose fuel cell equipped with an ion-exchange membrane as electrolyte.

Ever since the first designed prototype [2] was tested, generating 3.4 μW/cm^2^ stable power exclusively from the glucose present in the device’s environment, the possibility of envisaging the use of fuel cells as power sources for implantable devices has generated a lot of research [3,4,5,6,7,8,9]. Different types of glucose fuel cell exist, including microbial [4], enzymatic [5], or abiotic glucose fuel cells [6]. The abiotic glucose fuel cell (AGFC) uses abiotic catalysts to catalyse the glucose oxidation, and an ion-exchange membrane separates both anode and cathode compartments. Figure 1 shows the typical schematic structure of an AGFC.

An AGFC generates energy via the process of oxidation glucose, and in this process, the ion-exchange membrane, which has biocompatibility as its main characteristic, is shown to be absolutely essential. The membrane for AGFCs generally has two main functions: using an electrolyte as an ionic conductor and as separator of cathode and anode. When glucose and oxygen are mixed in the biological fluids, for the device to generate electricity, it is necessary that the anode and cathode of the battery work in separate chambers, thus facilitating the unidirectional flow of ions. Thus, oxidation and reduction reactions occur efficiently, and possible short circuits are avoided. The aforementioned ion-exchange membranes are, therefore, a fundamental part of the device. The properties of the membrane determine, to a great extent, the process performance; therefore, it is crucial to select the best choice for a given application. Although there are other alternatives, the Nafion membrane, which is a biocompatible cation-exchange membrane with excellent properties, is the most used. However, the influence of neutral molecules on membrane transport properties is still under investigation [10,11,12].

One crucial aspect is that the membrane remains invariant during its functional life; thus, the functionality of the complete equipment is not altered by changing its properties. This is a prerequisite for the reliable use of an implantable device. This study focused on a possible brain implant, which is integrated into the environment of the cerebrospinal fluid, from which it obtains its fuel. Exposure to this medium or other external agents can cause membrane changes, such as calcification due to the formation of calcium phosphate crystals, which causes certain properties, such as membrane permeability or ionic conductivity, to be altered. In certain cancer patients, radiation therapy is one of the most widely used treatments for brain metastases.

Even small amounts of radiation can induce significant changes in polymer properties, depending upon the chemical structure of a particular polymer [13]. Previous studies concluded that polymeric membranes and their properties can be affected by the incidences of different types of radiation [13,14,15,16,17,18]. In some cases, the impact of radiation has a sought-after effect on improving material performance. Thus, photo-irradiation can improve the performance of polymeric membranes in technical separations and other processes [18]. In [14], Nafion and SPEEK membranes were electron-beam irradiated to compare their radiation resistance properties. The results showed that Nafion demonstrated mechanical degradation, while SPEEK properties were improved. Studies about the impact of ultraviolet radiation on the performance of Nafion membranes showed that the radiation process led to dramatic changes in the membranes’ properties and nanostructure; nevertheless, an increase in the membrane proton conductivity was recorded [17]. However, medical applications require highly radiation stable materials, and a small or even insignificant effect of radiation on membrane properties would be desirable.

The aim of this study was to investigate the influence of ionizing radiations used in typical radiotherapy treatments on the properties of membranes used in glucose fuel cells. In particular, the work focused on the influence on the swelling properties of the membranes used as electrolytes in an abiotic glucose fuel cell. Swelling curves are usually used to characterize the swelling behavior of polymers [19]. Since the transport properties of the membrane are highly dependent on its swelling capacity and dimensional stability, an effect of radiation on these properties could affect the glucose fuel cell performance and, therefore, the implanted device’s functionality.

## 2. Materials and Methods

### 2.1. Materials

Four different types of commercial ion-exchange membranes were selected in this study. Some of their main characteristics are given in Table 1. Membrane Nafion 117 was selected for this study due it being a reference membrane typically used in membrane-based applications. We also selected another three membranes to carry out this study. One externally reinforced Nafion membrane and two internally reinforced Fumasep membranes, one cationic and one anionic, were used. The purpose of this study was to analyze the influence of the existence of a reinforcement in the membrane structure on the possible effect of the ionizing radiation on the membranes’ swelling properties. One anionic membrane was also selected to analyze the influence of membrane selectivity.

**Figure 2:**
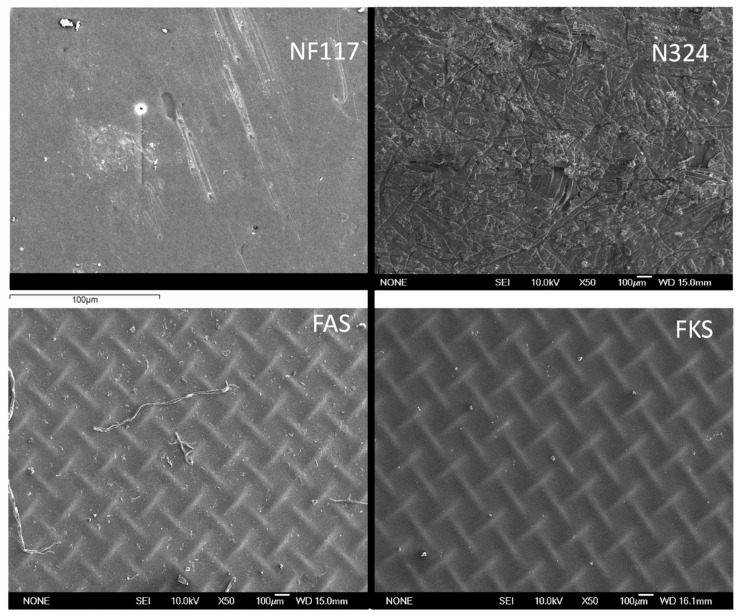
SEM images of membranes used in this work (Spanish National Centre for Electron Microscopy ICTS).

The Nafion 117 membrane (hereafter named NF117) and the Nafion N324 (hereafter named N324) were sourced from from Dupont de Nemours (USA). NF117, with a nominal equivalent weight of 1100 g/eq, is a homogeneous cation-exchange membrane consisting of a polytetrafluoroethylene backbone and long fluorovinyl ether pendant side chains regularly spaced, and is terminated through a sulfonate ionic group. There are no cross-links between the polymers. N324 (hereafter named N324) is a perfluorosulfonic acid cation-exchange membrane with a strong polytetrafluoroethylene fiber reinforcement. It is a bimembrane, with one layer having an equivalent weight of 1500 g/eq and a thickness of 0.025 mm, and the other layer having an equivalent weight of 1100 g/eq and a thickness 0.127 mm. A 24 × 24 strand per inch reinforcing fabric of PTFE was embedded, resulting in total thickness of 0.28 mm. Fumasep^®^ FKS–PET-75 (hereafter named FKS) and Fumasep^®^ FAS–PET-75 (hereafter named FAS) are standard grade ion-exchange membranes manufactured by FuMA-Tech GumbH (Germany). Both membranes are homogeneous and PET-reinforced membranes with a PEEK polymer matrix. The first membrane is a cationic membrane and the second membrane an anionic membrane. Figure 2 shows SEM (scanning electron microscope, Spanish National Centre for Electron Microscopy ICTS) images of the membrane samples used in this work. 

The solution used was an aqueous solution of NaCl 0.14 M and KCl 0.001 M as an electrolyte and glucose of 0.6 g/L as a non-electrolyte. This solution was selected so that the mixture solution had similar concentrations of ions and glucose as the cerebrospinal fluid (CSF) [20].

### 2.2. Methods

#### 2.2.1. Irradiation Treatment

An Elekta Versa HD linear accelerator (LINAC) with 6 MV FF photon energy and a RW3 I’m RT phantom was used to irradiate 5 samples of each type of membrane used by the Radiotherapy Service at the General University Hospital Gregorio Marañón in Spain. 

The RW3 phantom consisted of 18 1 centimeter-thick plates and 16 cm × 16 cm-sized water equivalent material at these energies. A Monte Carlo simulation using Elekta Monaco 5.11.02 treatment planning system (TPS) was performed to calculate the deposited dose at the membrane depth.

The experimental setup consisted of 9 RW3 plates for buildup and 9 plates for backscatter, with the 4 membrane samples located in between the plates at the radiation isocenter (100 cm). Five samples of different membrane materials were exposed to different radiation doses: 20 Gy, 30 Gy, 40 Gy, 50 Gy, and 60 Gy. These doses were selected based on usual central nervous system (CNS) treatments protocols employed at the hospital.

Usually, patients that need this type of treatment attend consecutive sessions of 2 Gy irradiation until they reach the total dose, following conventional protocols. However, the samples’ irradiation were performed in one session, for a time of around 40 min, due to self-reparation mechanism not being expected to appear, unlike in living cells [21].

#### 2.2.2. Thickness and Area 

Membrane thickness (*d*) was measured with a PCE-THM-20 material thickness meter with resolution 0.0002 mm. The final value of the membrane thickness was obtained by averaging the results of at least 10 measurements made at different points of the sample under study. The average values of the data and the corresponding standard deviation were calculated using the scientific data analysis and graphing SigmaPlot 11 software (Version 11). The maximum standard error was always lower than 0.001 mm. The area of the rectangular samples was measured by placing them over a graph paper with 0.5 mm resolution and measuring the length of both sides (*x*, *y*) of the samples. The area was then calculated as *A*= *x*·*y*.

#### 2.2.3. Swelling Degree

The swelling degree of the membranes after being submerged in solution was determined using the traditional gravimetric method. Before the experiments took place, the membranes were completely dried under vacuum. After that, they were weighted with a high precision balance $\left(\pm 0.0001\;g\right)$ and immersed in close bottles containing the mentioned solution. These bottles were maintained at 37 °C while the measurements were recorded. This temperature was selected in order to simulate the average temperature in the human body. From time to time after the immersion, the swollen membranes were taken out of the solution, wiped carefully with filter paper to remove the residual solution on the surface of the membrane, and weighed again. The difference between the original and the new measurement is the mass of the liquid gained by the membrane. This process was repeated in the following days to find out when the membrane was saturated, and its mass did not vary in the solution. The swelling degree at time *t* was calculated from the weight of the swollen and dry membrane samples according to the following expression:

(1) $$S\left(t\right)=\left(\frac{m\left(t\right)-m_{0}}{m_{0}}\right)$$

where *m_0_* and *m*(*t*) were, respectively, the masses of the dry and swollen membranes at time *t*.

#### 2.2.4. Dimensional Change

The dimensions of the membranes were measured in three spatial directions: *x*, *y*, and *z*. The two first dimensions, i.e., *x* and *y*, determine the membrane area, *A*, and the final one, i.e., *z*, corresponds to the membrane thickness, *d*. The percentage changes in area and thickness, due to the swelling process, were determined, respectively, from the expressions:

(2) $$EA\left(t\right)=\left(\frac{A\left(t\right)-A_{0}}{A_{0}}\right)\cdot 100$$

and:

(3) $$\;Ed\left(t\right)=\left(\frac{d\left(t\right)-d_{0}}{d_{0}}\right)\cdot 100$$

where *A*_0_ and *d*_0_ are, respectively, the area and thickness of the dry sample, while *A* and *d* are the area and thickness of the same sample after immersion in the solution for the time *t*. 

## 3. Results and Discussion

### 3.1. Influence of the Irradiation in the Dry Membranes

**Figure 3:**
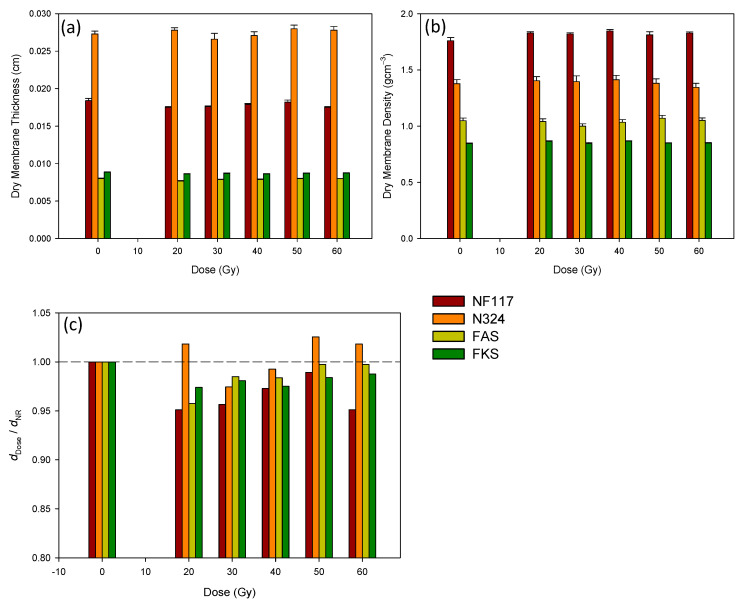
Thickness (**a**), density (**b**), and relative thickness of non-irradiated (NR) sample (**c**) of the dry membrane samples exposed at different doses.

The thickness (*z*), the *x* and *y* lengths, and the mass of all of the dry membrane samples were measured before the membranes were immersed in the solution. From these data, the densities of the dry membranes were also estimated. The results obtained for thickness and density are shown in Figure 3a,b for the different membranes and doses. In Figure 3c, the thickness relative to the dry sample thickness is also shown.

As can be observed in Figure 3a,c, the effect of the radiation on the thickness of the dry membranes was, in general, small, with the relative change always being less than 5%. A statistical analysis of the data indicated that there was not a correlation between membrane thickness and the received dose. With the exception of membrane N324, the radiation tended to decrease the membrane thickness with respect to the corresponding non-irradiated sample. The more affected membrane was the non-reinforced NF117. For this membrane, the radiation increased the density of the dry irradiated membranes by 3% for 50 Gy, and 5% for 40 Gy, with respect to the dry non-irradiated membrane. For the other membranes, however, the observed changes in the density due to the irradiation process were too small to discard as a possible cause of the same standard deviations in the measurements.

### 3.2. Influence of the Radiation in the Swollen Membranes

#### 3.2.1. Membrane Dimensions

**Figure 4:**
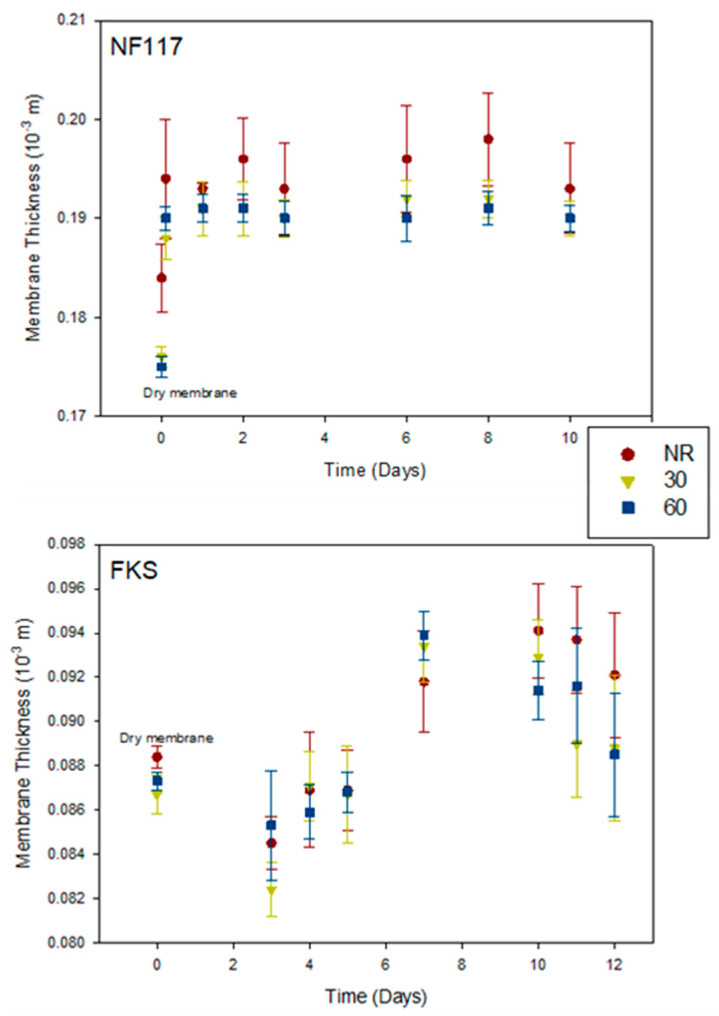
Thickness versus time for two of swollen membranes investigated at different irradiation doses: non-reinforced NF117 membrane (**above**); internally reinforced FKS membrane (**below**).

The evolution of the thickness of the swollen membranes over time was investigated. The membrane samples were submerged for 10–12 days in the solution. Figure 4 shows, as an example, the results obtained at different doses for two of the studied membranes: the non-reinforced NF117 membrane and the internally reinforced FKS membrane. In the figure, only the results obtained for the non-irradiated sample and the samples irradiated with 30 and 60 Gy are shown. In the Supplementary Materials, a figure outlining all doses can be found (Figure S1).

The membrane thickness was measured every day using at least 10 points from each sample. The points in Figure 4 indicate the corresponding mean values for the standard deviations. The values corresponding with the dry samples were also included for a better comparison. The observed behavior was different for Nafion membranes compared to Fumasep membranes.

**Figure 5:**
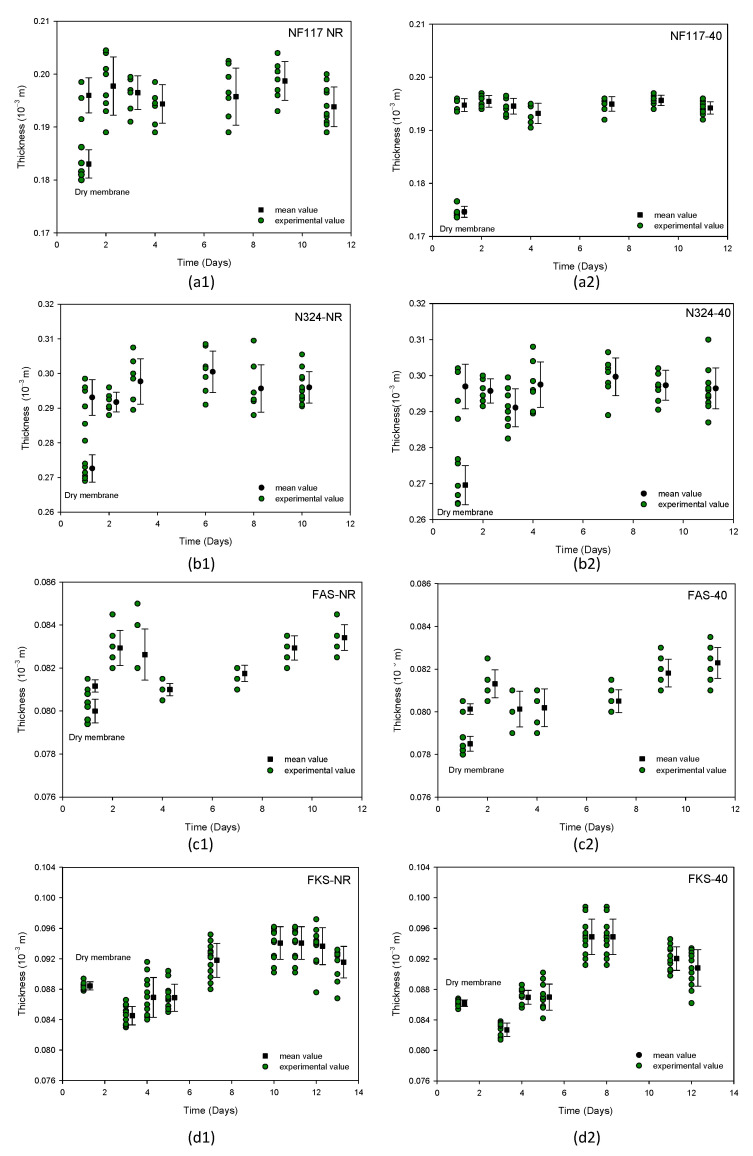
Thickness versus time of swollen membranes: (**1**) non-irradiated membranes; (**2**) irradiated with 40 Gy. (**a**) NF117; (**b**) N324; (**c**) FAS; (**d**) FKS.

For a given membrane, the behavior was qualitatively similar for all doses. Figure 5 shows, as an example, the time evolution of the thickness of the swollen membranes for the non-irradiated sample and the sample irradiated with 40 Gy. In Figure 5, green points correspond to experimental values, and the black points indicate the corresponding mean values with their standard deviations. The values for the dry sample (initial time is here considered as the day one) were also included for a better comparison The number next to the membrane name indicates the dose received, while NR denotes the sample not exposed to radiation.

As can be observed in Figure 4 and Figure 5, for Nafion membranes, the thickness increased when the membrane was placed in the solution, and it reached a value near the equilibrium value in the first days of the immersion. The externally reinforced Nafion membrane, i.e., N324, presented similar behavior to the N117 membrane. With the reinforced FKS membrane, the thickness decreased at the beginning, but later increased until reaching a maximum value and decreasing again; it did not reach a state of equilibrium until after 10 days of immersion in the solution. The anion-exchange membrane FAS showed different behavior. This membrane increased its thickness at the beginning of the process, later decreased, and finally reached an equilibrium value. As this change is due to the swelling process, the observed dynamic behavior must be related to the membrane-swelling kinetic. In the next section, we state that Fumasep membranes also showed a less defined trend regarding the swelling dynamic behavior. 

For a given membrane, the observed behavior was qualitatively similar for the non-irradiated and the irradiated samples. These results seem to indicate that the radiation did not influence the membrane thickness dynamic behavior during the swelling process. 

Table 2 shows the values of the swollen membranes thickness after 10 days in solution for the membranes and the doses. A correlation between membrane thickness and the received dose was not found. Moreover, it can be observed that the differences between the values of the samples irradiated with different doses and the value of the corresponding non-irradiated sample are, in general, within the experimental errors, which may be due to the scattering of thickness.

**Figure 6:**
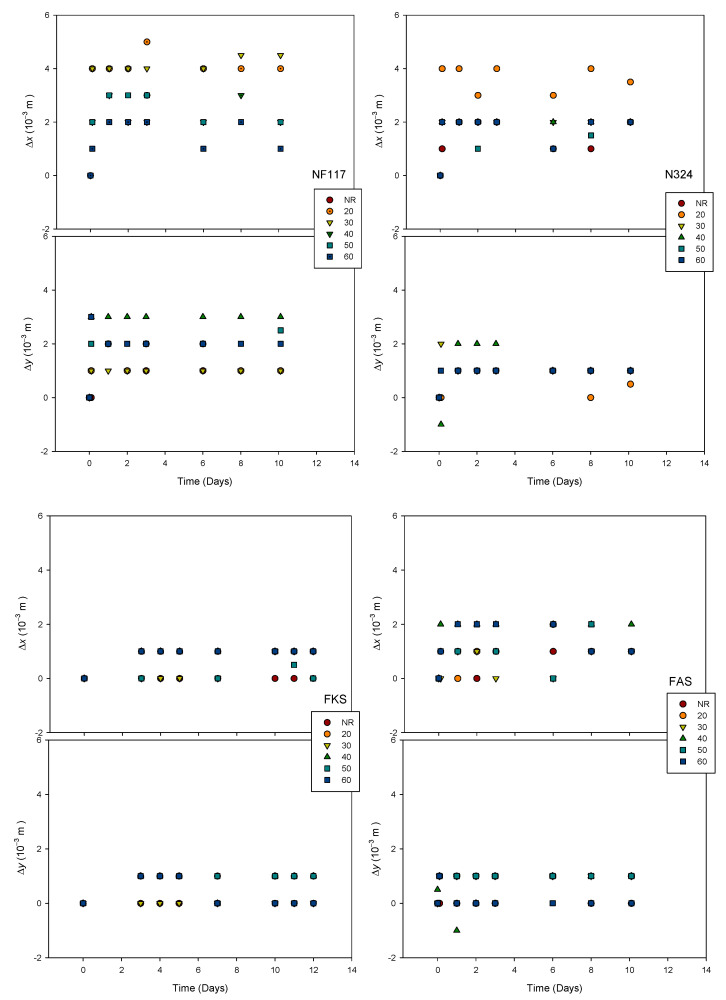
Dimensional changes due to swelling process in *x* and *y* lengths versus time for all investigated membranes. Lines are only visual guides. Experimental error for Δ*x* and Δ*y* was 0.7 mm.

Dimensional changes due to the swelling process in *x* and *y* lengths versus time are shown in Figure 6 for all of the investigated membranes and doses. The same *Y* axis scale was used in all cases for a better comparison. 

Changes between 0 and 4% were observed, depending on the membrane and dose. As can be observed, in all cases, the equilibrium state was reached after the first or second day. Although the influence of the received irradiation dose seems to be observed, a correlation with the received dose could not be established. The larger dimensional changes due to the swelling process were observed for the non-reinforced Nafion NF117 membrane, as was expected. Internally reinforced Fumasep membranes presented the lowest dimensional changes. For these membranes, according to the experimental error values, the dimensional changes would be negligible. The externally reinforced Nafion N324 membrane only showed a smaller change than the non-reinforced Nafion membrane in one of its directions. We noticed that both membranes showed an asymmetric dimensional change; thus, the membranes were deformed after the immersion process. The anisotropy may be due to the orientation of the polymer chains towards the direction of the cut for sampling [22].

**Figure 7:**
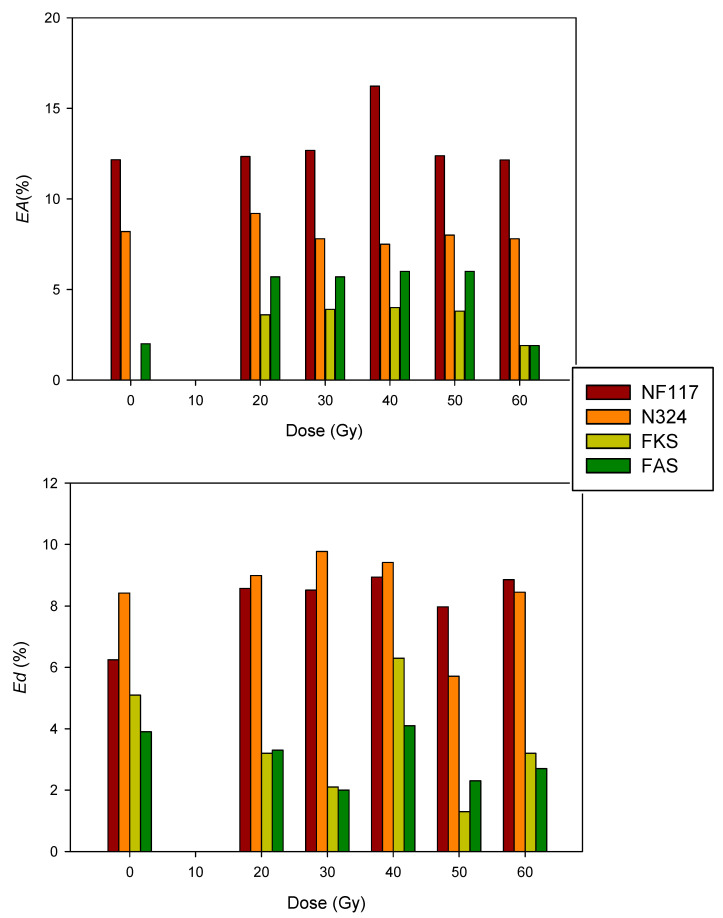
Area (*EA*) and thickness (*Ed*) relative change percentages due to swelling process as a function of dose for all investigated membranes.

Figure 7 shows the area and thickness change percentages due to the swelling process for all membranes and doses.

As can be observed, the area change in the non-irradiated samples was higher for the non-reinforced N117 membrane and lower for internally reinforced membranes. The non-irradiated FKS membrane did not show area change due to the swelling process. The N324 membrane, despite its external reinforcement, presented the highest thickness change. It is possible that this increase may be due to the layered structure of this membrane. These results may indicate that an internal reinforcement is useful to avoid dimensional changes due to the swelling process.

With respect to the influence of the radiation, in general, the effect on the area change was only significant for the membranes with internal reinforcement. Thus, irradiated FKS samples showed change in area different to that of the non-irradiated FKS sample. The thickness change in internally reinforced membranes was also affected by the irradiation process. With the exception of the samples irradiated with 40 GY, the irradiation process decreased the swelling thickness with respect to the non-irradiated sample. However, the non-reinforced NF117 membrane showed a higher effect of the radiation on the thickness change due to the swelling process. It is important because membrane thickness is a critical parameter in the transport properties in a membrane and could strongly affect the membrane performance in a given application. 

#### 3.2.2. Swelling Behavior

**Figure 8:**
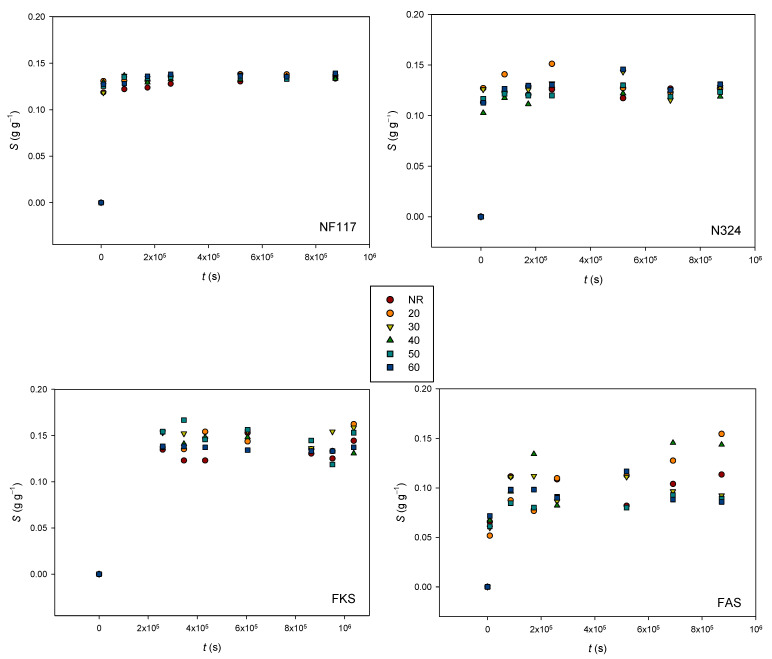
Swelling degree as a function of time for all membranes and doses.

The swelling degrees of all membranes samples were estimated according to Equation (1) at different days during the experiment. The obtained results are shown in Figure 8. The experimental error was always less than 4.0 × 10^−4^, 3.2 × 10^−4^, 1.6 × 10^−3^, and 1.4 × 10^−3^ gg^−1^ for NF117, N324, FKS, and FAS membranes, respectively. 

The obtained values were in accordance with typical values published for this type of membrane [23,24]. The general trend observed for all the membranes was that the swelling degree increased with time up to certain level, before leveling off and, finally, tending to an equilibrium value, i.e., *S*_eq_. However, the most stable trend was observed for the non-reinforced NF117 membrane. The externally reinforced Nafion N324 membrane showed more erratic behavior at first, before finally showing a more defined trend, which was similar that of the non-reinforced membrane. The membranes with internal reinforcement, especially the anionic membrane FAS, were those that showed a less defined trend, with a greater dispersion of values over time. 

Table 3 shows the mean values obtained from the values of the last two or three days for all membranes and doses.

We can observe that cation-exchange membranes showed similar degrees of swelling. The lowest value corresponded to the anion-exchange membrane FAS. When both Nafion membranes were compared, we observed that external reinforcement lead to a lower solvent content. If we take into account the fact that for internally reinforced membranes, a lower change dimensional was observed, these results seem to indicate that these membranes can swell a larger quantity of water with a lower dimensional change.

With respect to the influence of radiation, the results in Figure 8 show that the swelling degree of Nafion membranes was scarcely affected by the radiation, indicating that the radiation would not affect the swelling behavior of these membranes. Although significant relationships between dose and swelling degree were not found, a larger influence was observed for internally reinforced membranes. The water absorbed using an ion-exchange membrane is the result of a balance between the internal osmotic pressure due to the presence of ionic groups, contra-ions, and electrolytes absorbed in the polymer phase and the forces associated with the matrix elasticity [25]. The observed results indicate that the radiation affects the membranes’ internal reinforcement. Further studies would be necessary, however, to fully analyze this behavior. 

In order to determine the mass change in the membranes due to the swelling process, the membrane samples were taken out of the solution after the process, dried, and weighed once again. The mass change percentage was then calculated according to the expression:

(4) $$MC=\left(\frac{m_{0}^{\prime }-m_{0}}{m_{0}}\right)\cdot 100$$

where *m*_0_ is the initial mass of the dry membrane, and $m_{0}^{\prime }$ is the final mass of the membrane dried after the swollen process. The results are shown in Table 4.

Data in Table 4 show that the mass of dry membranes decreased after immersion in the solution, with this occurring independently of the received irradiation dose. This decrease was, in general, small for the Nafion membranes, being around 1%. However, the Fumasep non-irradiated membranes presented higher mass loss, especially the anion-exchange membrane, which had a loss of around 8–9%. 

We found that this mass loss was not observed in similar experiments using pure water with any one of the non-irradiated samples. Some studies showed that the presence of Na^+^ affects the intramembrane transport properties [26,27]. Madhav et al. [26] used accelerated degradation via Fenton´s test to examine the influence of Na^+^ presence in membrane degradation. They observed that the presence of NaCl accelerated the degradation of Nafion membranes in salty environments. They found that Nafion membranes produced C=O bonds during the degradation process, suggesting that the degradation of Nafion membranes could occur via main-chain unzipping and side-chain scission in the presence of hydroxyl radical, thus generating these bonds. For this reason, we think that the mass change observed may be attributed to a membrane degradation due to the presence of NaCl in the solution. The results found in this work would indicate that Fumasep membranes, especially the anion-exchange membrane, were the most degraded membranes in the swelling process. This different behavior may be due to the different polymeric matrix in both membranes—PTFE for Nafion membranes and PEEK for Fumasep membranes. As regards the effect of the irradiation, Nafion membranes did not experience a significant effect of the radiation on the mass loss. In contrast, for Fumasep membranes, the effect of the radiation seemed to reduce the mass loss due to the swelling process, although no correlation between the mass loss and dose was observed. This influence seems to be larger for the cationic FKS, which presented similar values for the mass loss as Nafion membranes after the irradiation process. These results could indicate that, in addition to the polymeric matrix, the type of fixed ion group would also have a key role in influencing this behavior. However, further work is necessary to gain a better understanding of these results.

Different adsorption kinetic models were used to model the swelling kinetic of polymer films and hydrogels [19,28,29,30]. We checked if the swelling process of the investigated membranes obeyed a second-order kinetic and monitored the effect of the radiation on the dynamic swelling behavior. If we assume that the swelling process of the studied membranes follows a second order kinetic model, the swelling rate at any time *t* may be expressed as [19]:

(5) $$S=\frac{k_{2}S_{eq}^{2}t}{1+k_{2}S_{eq}t}$$

which can be linearized to obtain the Schott´s equation [26]

(6) $$\frac{t}{S}=\frac{1}{k_{2}S_{eq}^{2}}+\frac{t}{S_{eq}}$$

**Figure 9:**
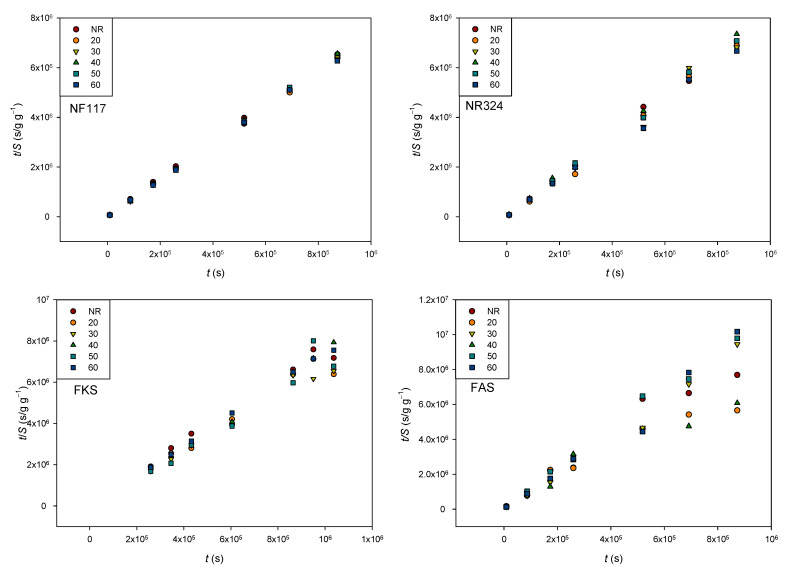
Plots of *t*/*S* vs. time for all membranes and doses.

According to Equation (6), the relation between *t*/*S* and *t* would be linear if the swelling process of the membrane followed a pseudo-second order kinetic, and the degree of swelling at equilibrium could be obtained from the slope of the corresponding straight line. Figure 9 shows the straight lines for all samples. 

From Figure 9, it is observed that for the Nafion membranes, the experimental data fit Equation (6) over the whole range of values. For these membranes, the influence of the radiation was very small. However, for the Fumasep membranes, the linear trend was only observed during the first days, especially for the anion-exhange FAS membrane. This last membrane presented the higher mass loss due to the swelling process, which could indicate that the degradation of the membrane would affect the membrane-swelling kinetic. For internally reinforced membranes, it was observed that the influence of the radiation was stronger, while the experimental values deviated from linear behavior as the time of immersion in the dissolution increased.

**Figure 10:**
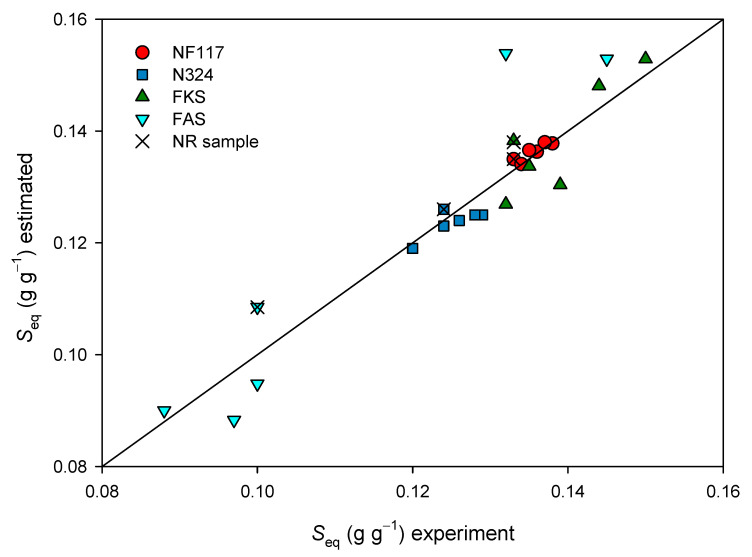
Estimated equilibrium swelling degree vs. experimental equilibrium swelling degree for all membranes and doses. Cross symbols indicate values corresponding to non-irradiated samples.

A value of the equilibrium swelling degree was estimated as fitting the experimental data to Equation (6). The estimated results are shown in Figure 10 and compared to the corresponding experimental equilibrium swelling degree values. 

As was previously shown, the data fit better for Nafion membranes. With these membranes, correlation r coefficients higher than 0.99 were obtained for all of the irradiation doses. With Fumasep membranes, the values of r were, in general, lower than 0.99, although they were always higher than 0.97. Although the difference between both values were within the experimental error, the results presented in Figure 10 may indicate that a pseudo-second order kinetic would not be valid for internally reinforced membranes, acting independently of the irradiation process.

## 4. Conclusions

The possible effects of ionizing radiation were studied for four commercial membranes that are typically used as electrolytes in glucose fuel cells to supply energy to a huge variety of implantable medical devices. For this purpose, membranes were irradiated with typical doses of a conventional radiotherapy treatment, simulating regular conditions of the biological working environment. The influence on the dimensional changes and swelling degree was analyzed as a function of the membrane structure and irradiation dose.

The results obtained showed that the effect of the radiation on the thickness of the dry membranes was, in general, small, with a relative change always being less than 5%. A statistical analysis of the data indicated that there was not a correlation between membrane thickness and the received dose. With the exception of externally reinforced membrane N324, the radiation tended to decrease the membrane thickness with respect to the corresponding non-irradiated sample. The more affected membrane was the non-reinforced NF117. For this membrane, the radiation increased the density of the dry irradiated membranes by 3% for 50 Gy, and by 5% for 40 Gy, with respect to the dry non-irradiated membrane. For the other membranes, however, the observed changes in the density due to the irradiation process were too small and could be discarded as a possible cause of the same standard deviations in the measurements. 

The swelling properties of the membranes were also analyzed. Membranes without reinforcement or with external reinforcement showed higher dimensional changes as a consequence of the swelling process, regardless of whether the membrane was irradiated. 

The results obtained showed that the radiation the membranes swelling dimensional changes affected in a different way depending on the membrane structure. Thus, the radiation mainly affected the area change in internally reinforced membranes and the thickness change in the non-reinforced membranes. Externally reinforced N324 membrane showed the lowest influence of radiation on the dimensional changes. 

The swelling degrees of Nafion membranes were scarcely affected by the radiation, indicating that the radiation would not affect the swelling behavior of these membranes. A larger influence was observed for internally reinforced membranes, although significant relationships between dose and swelling degree were not found. The observed results could indicate that the radiation affects the membrane internal reinforcement, although further studies would be necessary to analyze this behavior. 

The swelling process in Nafion membranes obeyed a second-order kinetic during the entire swelling process. Internally reinforced Fumasep membranes only showed this trend at the beginning of the swelling process. The latter membranes presented a higher mass loss after the immersion process and a higher influence of the received irradiation dose on the dynamic swelling behavior. 

Regarding the use of a membrane as an electrolyte in a glucose fuel cell, the more important parameters would be the thickness and the swelling degree. The first factor determines the pass of substances through the membranes, and the second factor strongly affects the ionic conductivity of the membrane and, therefore, the fuel cell performance. In the light of the results obtained, the influence of radiation over the investigated membranes was, in general, small, and the externally reinforced Nafion membrane seemed to be the membrane least influenced by the radiation process.

## Figures and Tables

**Table 1:** Some physical-chemical properties of membranes used in this work, as given by manufacturer.

Membrane	NF117	N324	FKS	FAS
Type	Cationic	Cationic	Cationic	Anionic
Reinforcement	No	PTF	Polyester	Polyester
Thickness (10^−6^ m)	183	152/280	74–87	72–85
IEC (meq g^−1^)	0.90	0.92	1.0	1.12
Fixed group	${-\mathrm{SO}}_{3}^{-}$	${-\mathrm{SO}}_{3}^{-}$	${-\mathrm{SO}}_{3}^{-}$	${-\mathrm{NR}}_{3}^{+}$
Provided ionic form	H^+^	H^+^	H^+^	Br^-^
Basic weight (mg·cm^−2^)	36	48	8.1–9.1	9.8–12.5

**Table 2:** Thickness of swollen membranes after 10 days in solution and corresponding standard deviations as a function of received irradiation dose for all membranes.

	Membrane			
Dose (Gy)	NF117	N324	FKS	FAS
0	0.198 ± 0.005	0.296 ± 0.004	0.092 ± 0.003	0.083 ± 0.006
20	0.191 ± 0.002	0.305 ± 0.006	0.086 ± 0.003	0.079 ± 0.001
30	0.192 ± 0.002	0.291 ± 0.006	0.087 ± 0.003	0.081 ± 0.006
40	0.196 ± 0.001	0.296 ± 0.006	0.093 ± 0.003	0.082 ± 0.007
50	0.197 ± 0.002	0.294 ± 0.004	0.086 ± 0.002	0.082 ± 0.007
60	0.191 ± 0.002	0.302 ± 0.004	0.088 ± 0.003	0.082 ± 0.004

**Table 3:** Equilibrium swelling degree (*S*_eq_) estimated as a mean value of values corresponds to last two or three days in solutions, as well as corresponding standard errors, as a function of received irradiation dose for all membranes.

	Membrane			
Dose (Gy)	NF117	N324	FKS	FAS
0	0.133 ± 0.003	0.124 ± 0.005	0.133 ± 0.010	0.100 ± 0.016
20	0.138 ± 0.001	0.126 ± 0.003	0.144 ± 0.016	0.132 ± 0.021
30	0.136 ± 0.001	0.129 ± 0.002	0.150 ± 0.012	0.100 ± 0.010
40	0.134 ± 0.003	0.120 ± 0.002	0.132 ± 0.012	0.145 ± 0.013
50	0.135 ± 0.003	0.124 ± 0.006	0.139 ± 0.018	0.088 ± 0.007
60	0.137 ± 0.002	0.128 ± 0.004	0.135 ± 0.023	0.097 ± 0.017

**Table 4:** Mass change percentage (*MC*) estimated according to Equation (4) for all membranes after swelling process.

	Membrane			
Dose (Gy)	NF117	N324	FKS	FAS
0	−1.28 ± 0.03	−1.11 ± 0.03	−3.64 ± 0.15	−9.06 ± 0.13
20	−1.25 ± 0.03	−1.27 ± 0.03	−0.93 ± 0.15	−7.59 ± 0.13
30	−1.29 ± 0.03	−1.25 ± 0.03	−1.99 ± 0.15	−8.47 ± 0.13
40	−1.30 ± 0.03	−1.33 ± 0.03	−1.03 ± 0.15	−7.94 ± 0.13
50	−1.44 ± 0.03	−0.92 ± 0.03	−1.46 ± 0.15	−8.47 ± 0.13
60	−1.15 ± 0.03	−1.05 ± 0.03	−0.81 ± 0.15	−8.33 ± 0.13

## Data Availability

Not applicable.

## Supplementary Materials

The following supporting information can be downloaded at: https://www.mdpi.com/article/10.3390/membranes13060592/s1, Figure S1: Thickness versus time for two of the swollen membranes investigated at all irradiation doses. Non- reinforced NF117 membrane (above). Internally reinforced FKS membrane (bellow).
