# Supplementary material for: Examining the Effect of Ionizing Radiations in Ion-Exchange Membranes of Interest in Biomedical Applications
Source: Membranes (Basel). 2023 Jun 10;13(6):592. doi: 10.3390/membranes13060592 (PMC10300967; doi:10.3390/membranes13060592)
Supplement: Supplementary file 1 [file membranes-13-00592-s001.zip › membranes-2407093-supplementary.pdf]

Supplementary Material for

Examining the effect of ionizing radiations in ion-exchange membranes of interest in biomedical applications.

Íñigo Lara[1], Yago Freijanes[2], Sagrario Muñoz[1], Gema Ruiz[2], and V. María Barragán[1,]*

[1] Department of Structure of Matter, Thermal Physics and Electronics; Faculty of Physics, Complutense University of Madrid, Spain, inigolar@ucm.es (I.L.); smsm@ucm.es (S.M.)
[2] Radiotherapy Service at the General University Hospital Gregorio Marañón, Madrid, senajiert@gmail.com (Y.F.); gruizg@salud.madrid.org (G.R.)
*Corresponding vmabarra@ucm.es

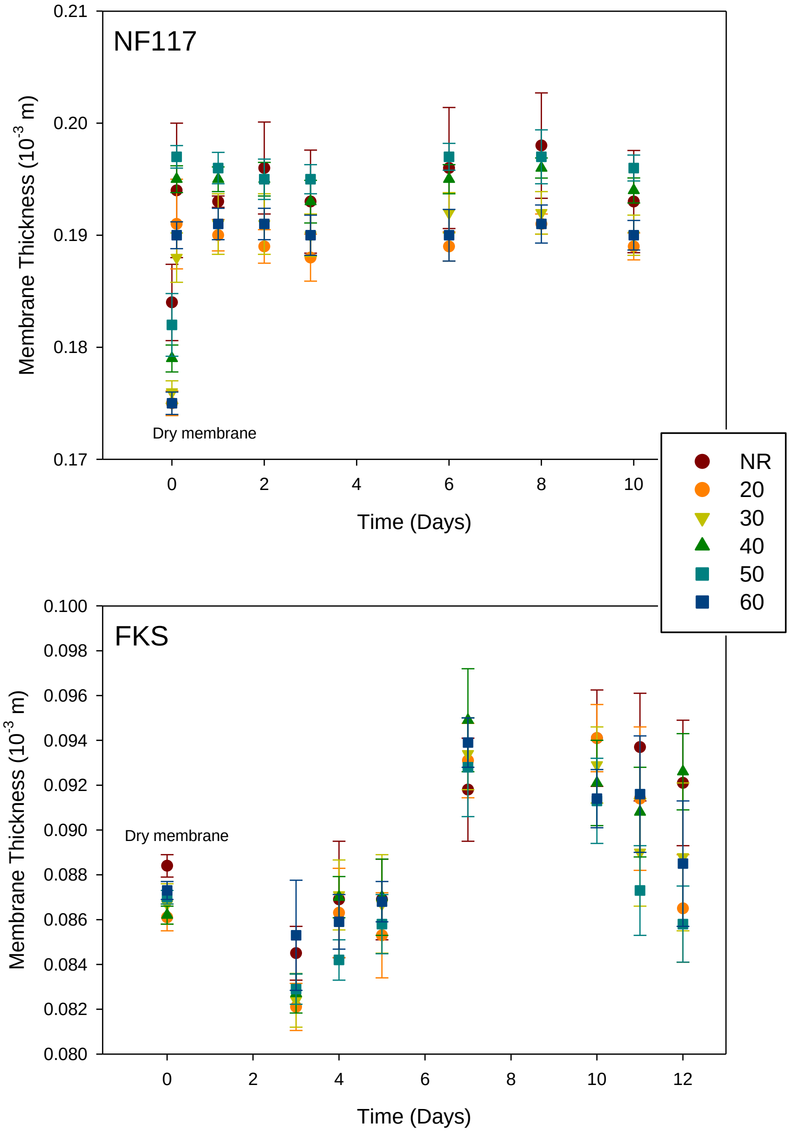

Figure S1. Thickness versus time for two of the swollen membranes investigated at all irradiation doses. Non- reinforced NF117 membrane (above). Internally reinforced FKS membrane (bellow).
